# Effects of metabolic memory on inflammation and fibrosis associated with diabetic kidney disease: an epigenetic perspective

**DOI:** 10.1186/s13148-021-01079-5

**Published:** 2021-04-21

**Authors:** Wen Zheng, Jia Guo, Zhang-Suo Liu

**Affiliations:** 1 Department of Nephrology, The First Affiliated Hospital of Zhengzhou University, Zhengzhou, People’s Republic of China; 2 Research Institute of Nephrology, Zhengzhou University, Zhengzhou, People’s Republic of China; 3 Key Laboratory of Precision Diagnosis and Treatment for Chronic Kidney Disease in Henan Province, Zhengzhou, People’s Republic of China; 4 Core Unit of National Clinical Medical Research Center of Kidney Disease, No. 1, Jianshe East Road, Zhengzhou, 450052 Henan Province People’s Republic of China

**Keywords:** Epigenetics, Metabolic memory, Diabetic kidney disease, Histone modification, Noncoding RNA

## Abstract

Diabetic kidney disease (DKD) is one of the most common microvascular complication of both type 1 (T1DM) and type 2 diabetes mellitus (T2DM), and the leading cause of end-stage renal disease (ESRD) worldwide. Persistent inflammation and subsequent chronic fibrosis are major causes of loss of renal function, which is associated with the progression of DKD to ESRD. In fact, DKD progression is affected by a combination of genetic and environmental factors. Approximately, one-third of diabetic patients progress to develop DKD despite intensive glycemic control, which propose an essential concept “metabolic memory.” Epigenetic modifications, an extensively studied mechanism of metabolic memory, have been shown to contribute to the susceptibility to develop DKD. Epigenetic modifications also play a regulatory role in the interactions between the genes and the environmental factors. The epigenetic contributions to the processes of inflammation and fibrogenesis involved in DKD occur at different regulatory levels, including DNA methylation, histone modification and non-coding RNA modulation. Compared with genetic factors, epigenetics represents a new therapeutic frontier in understanding the development DKD and may lead to therapeutic breakthroughs due to the possibility to reverse these modifications therapeutically. Early recognition of epigenetic events and biomarkers is crucial for timely diagnosis and intervention of DKD, and for the prevention of the progression of DKD to ESRD. Herein, we will review the latest epigenetic mechanisms involved in the renal pathology of both type 1 (T1DN) and type 2 diabetic nephropathy (T2DN) and highlight the emerging role and possible therapeutic strategies based on the understanding of the role of epigenetics in DKD-associated inflammation and fibrogenesis.

## Introduction

Diabetic kidney disease (DKD) is among the most common and severe complications of diabetes mellitus (DM). Type 1 diabetes (T1DM) is mainly caused by chronic autoimmune injury to pancreatic β cells, leading to an absolute deficit of insulin [1]. In contrast, type 2 diabetes (T2DM) is a metabolic disorder characterized by chronic hyperglycemia secondary to insulin resistance. T2DM and its complications are classical diseases as a result of interactions between multiple genetic and environmental factors [2]. According to previous studies, about 30% of patients with T1DM and 20% of patients with T2DM would ultimately be followed up with diabetic nephropathy (DN). Recently, due to the increased prevalence of DKD, this complication has emerged as a primary cause of end-stage renal disease (ESRD) [3]. The pathologic features of DKD are intricate and include thickened glomerular basement membrane, increased mesangial matrix, tubular interstitial fibrosis, and loss of podocytes [4]. Intuitively, inflammation and the resulting fibrosis are two major features of DKD. Renal fibrosis is a common complication of DKD and the leading cause of ESRD.

Hyperglycemia is a major risk factor for DKD. Many clinical studies have shown that intensive glycemic control in diabetic patients can reduce the incidence and progression of DKD [5, 6]. However, many diabetic patients still develop DKD despite receiving intensified glycemic control. The phenomenon of hyperglycemic "metabolic memory" of DKD refers to the observation that diabetic patients are prone to develop diabetes-related complications due to early hyperglycemia, even if effective measures were taken to control blood glucose and maintain it within normal levels in the later stage of the disease. The underlying mechanisms of "metabolic memory" include a range of pathogenic factors associated with the diabetic complications, such as the advanced glycation end products (AGEs), oxidative stress, inflammation and epigenetic modifications [7, 8]. Among these pathogenic factors, epigenetics has emerged as "link" between metabolic memory and DKD development and progression, which has attracted the attention of the medical community.

Epigenetics refers to the changes of gene expression and function and the generation of heritable phenotype without alteration of DNA sequences [9]. Actually, the term “epigenetics” was first coined by Conrad Hal Waddington in 1942. It is believed that genotypes could determine different phenotypes through some accidental or unknown mechanisms. Based on our understanding of these relations, epigenetics and heredity can be considered close relatives [10]. Epigenetic changes primarily include DNA methylation, histone modifications, and non-coding RNA [11]. Emerging studies suggest a key role of epigenetics in the regulation of physiological and pathological processes associated with DKD development and progression including renal fibrosis, chronic inflammation, and other pathologic features [12, 13]. The present review will focus on discussing the mechanisms of metabolic memory, and its relationship with renal inflammation and fibrosis associated with DKD based on recent epigenetic perspectives, and thus highlight some possible epigenetic therapeutic strategies for DKD treatment.

### Pathogenic mechanisms accounting for DKD

When progressed to DN, whether T1DM or T2DM, the histologic changes of the kidneys are similar. Multiple mechanisms are involved in the development of both T1DN and T2DN, which include metabolic disorders, such as hemodynamic changes induced by hyperglycemia, podocyte injury caused by oxidative stress, autophagy and apoptosis, renin-angiotensin system activation, infiltration of inflammatory cells and mediators, and accumulation of AGEs [14–17]. Chronic inflammation has been recognized as a promoting factor for the development of DKD to ESRD [18]. Additionally, chronic and relentless fibrosis in both glomerular and tubulointerstitial compartments is another notable and reliable indicator of the progression of DKD [19].

### Role of renal inflammation in DKD

The inflammatory response can be driven by a variety of mechanisms, including activation of macrophages and accumulation of immune cells in the kidneys. Through the leukocyte adhesion molecule, including vascular cell adhesion molecule 1 (VCAM1) and intercellular adhesion molecule 1 (ICAM1), monocytes can adhere to endothelial cells and migrate through endothelial cells by secreting chemokines [20]. The chemokine (C–C motif) ligand 2 (CCL2), which represents the initial stage of glomerular and tubular inflammation, can induce the recruitment, migration, and adhesion of inflammatory cells following DKD-associated tissue injury [18]. In the kidneys of diabetic animal models and DKD patients, macrophages tend to infiltrate the kidney tissue upon the upregulation of chemokines. The accumulation of macrophages in the kidneys is a prominent feature of the progression of chronic kidney diseases [21] and is closely associated with the decline in glomerular filtration rate and poor prognosis [22]. The deletion of macrophage scavenger receptor protects DM mice from proteinuria, glomerular hypertrophy, and transforming growth factor-β (TGF-β) overproduction [23]. In turn, tumor necrosis factor-α (TNF-α) secreted by activated macrophages can stimulate the production of CCL2 in kidney cells. A recent cohort study [24] also found that multiple inflammatory proteins are involved in the terminal development of DKD and can improve the risk assessment of DKD as novel biomarkers.

The high glucose (HG) environment in the tissues of DM patients can also activate multiple cascades signaling pathways to coordinate cellular transcription, thereby induce monocyte infiltration, chemokines production and secretion, which drive inflammation, and macrophage infiltration leading to tissue damage. The Janus kinase/signal transducers and activators of transcription (JAK-STAT) signaling pathway play an important role in renal cells, including mesangial cells (MCs), podocytes, and tubular epithelial cells [25]. Zhang et al. [26] showed that the overexpression of JAK2 in podocytes can lead to increased kidney damage in T1DM mice. Additionally, the NF-E2-related factor 2/Kelch-like ECH associated protein 1 (Nrf2-keap1) pathway activation improves pathological changes in glomeruli of diabetic mice injected with streptozocin(STZ)[27]. Nrf2 inhibits the inflammatory response by directly regulating the transcription of pro-inflammatory factors such as interleukin (IL) -1 and IL-6. Moreover, downstream targets of NF-κB include adhesion molecules and pro-inflammatory cytokines (e.g., IL-6, TNF-α, CCL2), which all contribute to the development of DKD [28].

### Role of renal fibrosis in DKD

The accumulation of extracellular matrix (ECM) proteins, such as type I and type III collagen, fibronectin, and laminin in the tubulointerstitial and mesangial areas is another key feature of DKD [29]. During the early stages of DKD, renal biopsy shows the accumulation of ECM proteins in the mesangial space, which gradually leads to the appearance of glomerular sclerosis [30]. The continuous protein synthesis increased deposition and decreased degradation of these ECM proteins, and their post-translationally modified isoforms contribute to the progression of fibrosis [31]. In the late stages of diabetic glomerular sclerosis, the deposition of type I and type III collagen increases dramatically [32]. Thus, the accumulation of ECM proteins plays a vital role in throughout all stages of DKD renal fibrosis. Moreover, TGF-β signaling pathway acts as a profibrotic factor, promoting renal fibrosis. TGF-β promotes the expression of downstream ECM proteins and their regulatory factor plasminogen activator inhibitor-1 (PAI-1), which is the main substance that inhibits cytoplasmic degradation, mainly by activating the transcription factors Smad2-4 [33, 34]. Connective tissue growth factor (CTGF) activation acts as a downstream mediator of fibrotic activity of TGF-β1, which increases expression of fibronectin and type IV, III and type I collagen, and promotes the deposition and assembly of ECM proteins [35, 36]. Therefore, reversing the expression of these fibrosis regulatory genes or ECM proteins may have potential renal protective effects against DKD.

### Metabolic memory and epigenetic regulation in DKD

Nowadays, during the early stage of T1DM or T2DM, long-term intensive glycemic control is recognized as an effective preventive measure against diabetic complications, especially DKD development [37, 38]. The evidence was undoubtedly derived from a series of clinical trials, such as Diabetes Control and Complications Trial (DCCT) and epidemiology of diabetes interventions and complications (EDIC) where the term “metabolic memory” was first introduced. Between 1983 and 1989, 1441 patients with T1DM were recruited and randomized to receive intensive or conventional treatment. The difference of average glycated hemoglobin (HbA1c) between DCCT intensive treatment group and conventional treatment group was about 2% (7% vs. 9%). Based on a period of 20 years’ follow-up, the results of EDIC study demonstrated that despite the HbA1c of two groups were maintained within similar levels, the overall mortality rate of patients in the routine treatment group was higher than that of the general population [5]. Additionally, 3867 newly diagnosed type 2 diabetic patients were enrolled by the UK Prospective Diabetes Study (UKPDS) [6]. In this 15-year prospective study, the results also demonstrated that early intensive glycemic compliance can lead to a long-term kidney benefit. The phenomenon of metabolic memory has been termed “legacy effect” by the UKPDS investigators.

Metabolic memory, or legacy effect, refers to the "memory" effect of the body on early stage hyperglycemia, which plays an important role in the development of DKD [39]. The phenomenon of "metabolic memory" has also been confirmed in various experimental models. E1-Osta et al. [40] induced cultured aortic endothelial cells in vitro under transient hyperglycemic conditions and found that oxidative stress remained activate, and the high expression of inflammatory factors persisted even with the switch of the culture medium to normal glycemic levels. Additionally, a metabolic memory model of diabetes was successfully developed in zebrafish with 0.3% STZ solution [41]. The "metabolic memory" phenomenon has advanced our understanding of the mechanisms of diabetes complications including DKD. It has aroused people's interest in determining the potential molecular mechanisms of the metabolic memory of diabetes.

In recent years, modulation of epigenetic mechanisms has been linked to reduction of hyperglycemia and reversal of metabolic memory. Miao et al. [42] elucidated the histone modifications of peripheral blood lymphocytes and monocytes in patients with T1DM. They compared H3K9ac, H3K4me3, and H3K9me1 histone modifications in the DCCT conventional treatment group with the intensive treatment group and found a correlation between HbA1c and H3K9ac. Moreover, many genes with promoter regions recruited with high levels of H3K9ac histone modification are involved in the NF-κB pathway, which in part explains metabolic memory phenomena. Similarly, a study investigated epigenetic DNA methylation levels at specific genetic loci in patients with T1DM in the DCCT conventional and intensive treatment groups during the EDIC follow-up stage. A total of twelve annotated differentially methylated loci were discovered, including thioredoxin interacting protein (TXNIP). TXNIP has been shown to be associated with hyperglycemia and other related complications. Additionally, TXNIP-sustained hypomethylation can be induced by high glucose in cultured THP1 Monos in vitro [43]. The discovery of persistent memory embedded in these epigenetic features and the strong association with previous exposure to high glycemic conditions supports the potential role of epigenetics in metabolic memory of DKD.

### Epigenetic mechanisms involved in renal inflammation and fibrosis of DKD

Recently, the role of epigenetics in the pathology of diabetic kidney disease has been established. Covalent modifications of DNA and histones, such as histone acetylation, and non-coding RNAs could interfere with gene expression, through the promoter or enhancer regions, without altering the genetic sequence of the relevant gene [44]. Next, we will review how epigenetic changes occur, accumulate, and contribute to the development and progression of inflammation and fibrosis in DKD.

### DNA methylation

DNA methylation, which is considered a major transcriptional regulator, is the most widely studied epigenetic mechanism. DNA methylation is a process that occurs under normal physiological conditions, mediated by a series of DNA methyltransferases (DNMTs) [45]. This family of enzymes include DNMT1, DNMT3a, and DNMT 3b [46]. Conversely, DNA methylation can be reversed by a ten-eleven translocation (TET) protein that converts 5-methylcytosine to 5-hydroxymethylcytosine [47]. Several studies have linked high blood sugar and diabetic nephropathy to epigenetics. Although the genome-wide DNA methylation analysis surged in both T1DN and T2DN, the further studies of DNA methylation on renal inflammatory and fibrogenesis processes concentrate upon T2DN.

### Genome-wide DNA methylation analysis

Recent studies attempted to identify methylation changes associated with DKD and the role of site-specific methylation editing in DKD development. Most of these studies were performed in mice or alternative types of cells, such as cells isolated from blood or saliva samples [48, 49]. A study sequenced the methylation group map of human T1DN tubule samples found that a decrease in the level of cytosine methylation in TNF-α differentially methylated region (DMR) plays an important role in controlling TNF-α transcription levels [50]. In 181 Pima Indian type 2 diabetic patients, an epigenome-wide association study examined the association of cytosine methylation levels in 397,063 genomic CpG sites with estimated glomerular filtration rate (eGFR). The result showed that methylation levels at 77 sites were significantly associated with decreased eGFR, and 3 of these sites were significantly associated with human renal tissue fibrosis [51]. To determine whether changes observed in blood samples are also related to target tissue or cell type, a recent cross-sectional study of cytosine methylation in renal tubules samples from 91 type 2 diabetic and non-diabetic patients, as well as patients with varying degrees of kidney disease, confirmed that the methylation levels of 65 probes correlated with the degree of renal fibrosis [52]. Moreover, after treatment of MCs with TGF-β1, 5140 genes with significant differences were identified by Next-Generation Sequencing (NGS) identification. Among these genes, *Tnfrsf11b*, *Rxfp3*, *MMP9*, *Syn1*, *IL6*, and *Megf6* are known to be involved in the regulation of fibrosis and inflammatory pathway [53].

### DNA methylation involved in renal inflammatory processes

DNA methylation can modulate the inflammatory processes and the expression of inflammatory-related protein in DKD. However, the current studies mainly focus on T2DN. The level of DNA methylation key enzyme DNMT1 in T2DN patients increases with the increase of inflammatory activity in peripheral blood monocytes. Inhibition of DNMT1 by 5-Aza-2′-deoxycytidine significantly increased the proportion of CD4^+^, CD25^+^ regulatory T cells in peripheral blood mononuclear cells of early-stage T2DN animals. Additionally, aberrant cytosine methylation of mTOR upstream regulators, which is induced by upregulation of DNMT1 in diabetic immune cells, induces inflammation in diabetic kidneys [54]. The decrease in the methylation level of the promoter region of the angiogenin-like protein 2 (ANGPTL2), a pro-inflammatory circulating protein, also contributes to the development and progression of albuminuria in type 2 diabetic patients [55]. These studies suggest that high glucose-induced genomic DNA methylation may be a potential cause of DKD.

### DNA methylation involved in renal fibrotic processes

DNA methylation is also involved in the regulation of the fibrotic processes, which primarily revolves around T2DN as well. In db/db mice, the nuclear receptor pregnane X receptor (PXR) can play a role in fibrosis in the injured kidney by activating the response gene to complement 32 (Rgc32) [56]. Matrix metalloproteinases (MMP) are a group of peptidases involved in the degradation of ECM. A study of detecting the methylation level of the gene promoter region in peripheral blood of patients showed that the level of MMP-9 was lower in the DKD group than in the normal control group, and the simple diabetes group [57]. Moreover, the hypomethylation of *TIMP-2* and *AKR1B1* genes is associated with proteinuria in patients with early DKD, and TIMP-2 methylation levels are associated with increased fibronectin expression and ECM accumulation [58]. TGF-β1 stimulation also plays a regulatory role in renal fibrosis by regulating DNA methylation of various genes [53]. When TGF-β1 is stimulated, DNMT1-mediated hypermethylation of *RASAL1* can lead to increased fibroblast activation and fibrosis [59]. However, in STZ-induced DKD model, TET3-mediated methylolation reverses the methylation of the *RASAL1* promoter, leading to reversal of renal fibrosis [60]. Clinical studies have shown that there is a gradual decrease in DNA methylation in the TGF-β regulatory region in patients with DM and DKD. A recent study [61] also confirmed that the use of short hairpin RNA or oral inhibitors to inhibit TET2 expression can increase the methylation of CpG islands in the TGF-β regulatory region, meanwhile, significantly decrease ECM protein expression levels and mesangial cell proliferation.

### Histone modification

Histone post-translational modifications (PTMs) control chromatin relaxation and gene transcription. Studies have shown that persistent changes in histone modifications caused by hyperglycemia can affect the pathways involved in DKD. The covalent PTMs of nucleosome histones occurs mainly at the tail of amino acids, including lysine acetylation (Kac), methylation (Kme), and ubiquitination, arginine methylation and serine/threonine phosphorylation [62]. Among these different types of PTMs, histone Kac and Kme role in DKD has been well studied and will be discussed here.

### Common histone PTMs in DKD

Histone acetylation is primarily associated with gene activity and can promote gene transcription by neutralizing the positive charge of histone amino acid residues and attenuating the binding of histones to the negatively charged DNA [63]. In metabolic memory studies in T1DM patients, the level of histone acetylation was found to be associated with the past levels of HbA1c at earlier disease stages, which has a cumulative effect on H3K9 hyperacetylation at key genomic regions [42]. Histone acetylation plays a dynamic regulation of mutual antagonism between histone acetyltransferase (HATs) and histone deacetylase (HDACs). HKac is mediated by HATs, including P300, CBP, PCAF, and TIP60, which activate gene transcription by adding acetyl groups to conserved lysine. A study has shown that p300 is involved in upstream epigenetic mechanism of HG-induced endothelial oxidative stress, ECM protein gene expression and NF-kB signaling [64]. However, the process of deacetylation is mediated by HDAC 1–11 and Sirtuins (Sirt)1–7 [65]. Different classes of HDACs have also been shown to act as regulators in DKD pathophysiological processes such as inflammation and fibrosis.

Histone methylation, in contrast, participate in gene activation or inhibition depending on the extent of methylation and the amino acid residue modified. In general, methylation markers associated with gene transcriptional activation include K3K4me1/2/3, H3K36me2/3, and H3K79me2. Conversely, H3K9me2/3, H3K27me3, and H4K20me3 are usually associated with gene silencing or inhibition [63]. Like acetylation, the process of histone methylation is also catalyzed by enzymes. Histone methylation is mediated by histone lysine methyltransferase (HMTs) and cleared by histone lysine demethylase (HDMs). The FinnDiane Study Group has shown that the genes encoding methyltransferases SETD7, SUV39H1, and SUV39H2 are polymorphic, and the genetic variation of these genes has protective effects against diabetic microvascular complications [66].

### Histone modifications involved in renal inflammation

**Fig. 1:**
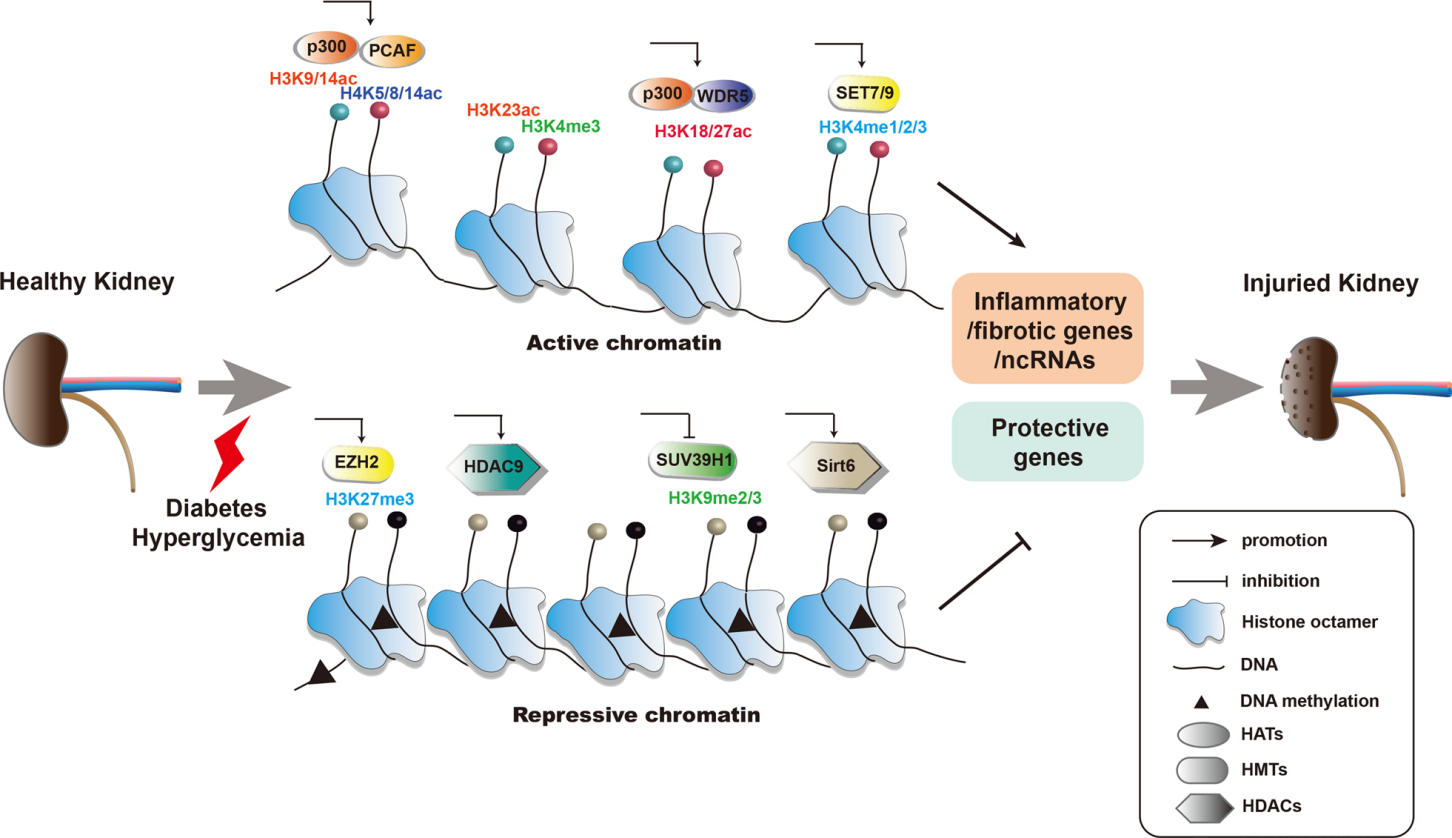
Epigenetic modifications of pathologic genes associated with DKD. In diabetic conditions, a series of epigenetic modifications occurred in protective genes, inflammatory and fibrotic genes, or even ncRNAs contribute to renal damage. In the formation of active chromatin, H3K4me1/2/3 mediated by histone methyltransferases such as SET7/9 and H3K9/14ac, H3K18/23/27ac, and H4K5/8/14ac mediated by histone acetyl transferases such as p300, WDR5, and PCAF are involved. In the contrary, H3K9me2/3 and H3K27me3 mediated by HMTs EZH2 and SUV39H1, respectively, and histone PTMs mediated by HDACs Sirt6 and HDAC9 play repressive role on the transcription of protective genes. DNA methylation mediated by DNA methyltransferases is also associated with transcriptional repression. Under disease state such as diabetes and renal injury, epigenetic alterations can also lead to the dysregulation of ncRNAs, which take part in persistent epigenetic alterations. Epigenetic modifications of pathologic genes associated with DKD play key roles in metabolic memory. HATs: histone acetyl transferases; HMTs, histone lysine methyltransferase; HDACs: histone deacetylase; PTMs, post-translational modifications

There is accumulating evidence for mechanisms by which histone modifications control DKD inflammation (Fig. 1). In blood mononuclear cells of both T1DM and T2DM patients, HG treatment can increase the recruitment of p300/CBP and PCAF at the promoters of inflammatory genes such as *TNF-α* and *COX-2*, with increased acetylation level of histone H3K9/14 and H4K5/8/12 [67]. In STZ-induced diabetic rat model, NF-κB also can recruit p300 to the promoter region of *iNOS* and induce H3K9ac [68]. However, in STZ-induced diabetic mouse model, the histone deacetylase Sirt6 inhibits *Notch1* and *Notch4* transcription by deacetylating histone H3K9 and exerts podocyte protection through anti-inflammatory and anti-apoptotic effects [69].

Studies of T2DN experimental models also support a role for histone modifications in renal inflammation. In diabetic db/db mice, HDAC9 silencing also reduces the release of inflammatory cytokine, podocyte apoptosis, and kidney damage [70]. Several studies have also revealed a link between chromatin histone lysine methylation and the expression of inflammation-related genes. Studies by Rama Natarajan et al. [71] and Wang et al. [72] have shown that both cultured cells and renal tissues of diabetic patients have been found to have decreased H3K9me3 concomitant with the silencing of HMT SUV39H1 at the promoters of the inflammatory genes *IL-6* and *MCP-1*, which resulted in the upregulation of the expression of these inflammatory genes. Endoplasmic reticulum (ER) stress can trigger the expression of *MCP-1* in the kidney of db/db mice induced by H3K4 methyltransferase SET7/9, which is associated with an increase in the active gene related chromatin marker, H3K4me1 [73]. The effect is also transiently present in high glucose-stimulated aortic endothelial cells. Transient or previous hyperglycemia leads to various methylation and demethylation events that allow the pro-inflammatory pathways to be continuously activated, which further confirms the role of “metabolic memory” in DKD [40]. Moreover, the administration of the H3K27 demethylase inhibitor GSK-J4 improves renal dysfunction and reduces inflammation, apoptosis, and DNA damage in diabetic db/db mice [74].

### Histone modification involvement in renal fibrotic processes

Glomerulosclerosis and tubulointerstitial fibrosis are key events in the pathogenesis of DKD. Many studies have found that histone modification participates in regulating the expression of ECM-related genes in T1DN and T2DN (Fig. 1). In kidneys of STZ-induced diabetic mice, MRTF-A can recruit p300 and WDR5 to bind to the collagen I promoter, and activate the transcription of the gene by increasing the transcription of H3K18/27ac and H3K4me3 to promote renal fibrosis [75]. Furthermore, several studies have shown that the expression of profibrotic genes such as *PAI-1*, *CTGF*, and *p21* can be regulated by PTMs in DKD [76]. TGF-β increased the recruitment of SET7/9 on the promoter regions of these genes, accompanied by increased level of chromatin markers associated with the active genes (H3K4me1, H3K4me2, and H3K4me3), and decline of repressed marker levels (H3K9me2 and H3K9me3) [13]. Similarly, the decrease of H3K9me2 is accompanied with the increased occupancy of H3K4me1/3 and SET7/9 on the p21 promoter, which promoted the expression of *p21* gene in HG-treated rat MCs. The effect can be reversed by TGF-β1 antibody [77]. In addition, TGF-β1 can recruit HATs CBP/p300 and increase the enrichment of H3K9/14ac in the promoter region to promote the genes expression of *PAI-1* and *P21* genes [78]. And the increased levels of H3K9/14ac and H3K18ac in HG-induced MCs augmented the expression of profibrotic factors CTGF and TGF-β1 [79]. Moreover, emerging evidences have demonstrated that PTMs also participate in renal fibrotic processes of T2DN models. Sayyed et al. [80] have demonstrated that glomerular sclerosis is associated with increased renal H3K9ac, H3K23ac, and H3K4me2 in advanced T2DN. Under hyperglycemia/hyperinsulinemia conditions, mRNA expression of fibrin 1 is elevated in the kidneys of diabetic db/db rats due to elevated levels of histone H3 acetylation [81].

### Histone PTMs-oriented therapies against DKD

Unlike methylation which is persistent and long-standing, histone acetylation is a reversible dynamic process [82]. This makes it possible to alleviate the progression of DKD by inhibiting HATs or HDACs. A series of small molecule inhibitors or drugs have been discovered in recent years in response to various mechanisms of inflammation and fibrosis regulated by histone PTMs in DKD (Table 1). However, the studies suggested that administration of histone PTMs inhibitors or drugs mainly work on T1DN. Curcumin can alleviate the increase expression of diabetic-induced ECM proteins in the kidney by inhibiting HAT p300 and its binding factor NF-κB [83]. C66, a curcumin analog, can inhibit the increase in H3K9/14ac levels and p300/CBP occupancy on gene promoters of *TCGF*, *PAI-1*, and *FN-1*, thus prevent diabetes-induced renal dysfunction [84]. In STZ-induced diabetic kidneys and cultured human proximal renal tubular epithelial cells, the HDACs inhibitor trichostatin A (TSA) has also been shown to block TGF-β1-induced EMT [85, 86]. Through HDAC inhibition, valproic acid cannot only significantly exert anti-inflammatory activity via reducing NF-κB [68], but it also improves kidney function by reducing damage and fibrosis [87]. In addition, some Chinese herbal active ingredient extracts, such as 2,3,5,4′-tetrahydroxysti-2-O-β-D-glucoside (TSG) and esculetin, have been found to attenuates alteration in *CTGF* and *MMP-13* gene expression by reducing changes in the acetylation and methylation of histone 3, thus ameliorate renal fibrosis in DKD [88, 89]. In summary, these aforementioned reports provide potential therapeutic possibilities for DKD.

**Table 1:** The administration and effect of histone PTMs regulators on DKD

Inhibitors category	Experimental models	Administration stage	Duration of treatment	Mechanisms	Ref
*HAT inhibitor*					
Curcumin	STZ-induced SD rats	DM	4 weeks	alleviate the increase expression of ECM proteins by inhibiting HAT p300 and its binding factor NF-κB	[83]
C66	STZ-induced mice	DM	12 weeks	inhibit the increase in H3K9/14ac levels and p300/CBP occupancy on gene promoters of TCGF, PAI-1, and FN-1	[84]
*HDAC inhibitor*					
TSA	STZ-induced SD rats	DM	4 weeks	Suppresses TGF-β1-induced epithelial-to-mesenchymal transition and activation of HDAC2	[85, 86]
Valproic acid	STZ-induced SD rats	DM	8 weeks	exert anti-inflammatory activity via reducing NF-κB and improve kidney function by reducing renal damage and fibrosis	[87]
*Drugs*					
TSG	STZ-induced SD rats	DM	8 weeks	inhibit oxidative stress, inflammatory, and expression of TGF-β1 partly mediated by activation of SIRT1	[88]
Esculetin	STZ-induced SD rats	DKD	8 weeks	attenuate alteration in Mmp13 and Bmp6 gene expression by involving change in acetylation and methylation of histone H3	[89]

### Modulation of non-coding RNAs

High-throughput sequencing technology has confirmed that less than 3% genes of the human genome can be transcribed into protein-encoding RNA, so the vast majority of RNA is non-coding RNA (ncRNAs) [90]. ncRNAs, which mainly include microRNAs (miRNAs), long non-coding RNAs (LncRNAs), and circular RNAs (CircRNAs), are once considered to be “transcriptional noise” in various diseases. However, with the in-depth research of RNA function, a growing evidence suggests that ncRNAs are involved in multiple physiological and pathological processes, including the pathogenesis of DKD [91]. As another major mechanism of epigenetic regulation, ncRNAs can influence gene expression through both transcriptional and post-transcriptional mechanisms and thereby regulate the progression of inflammation and fibrosis in DKD.

### The role of microRNAs in diabetes-related renal inflammation and fibrosis

MiRNAs, a class of endogenous single-stranded RNAs with a length of 20–22 oligonucleotides, are proved to be expression-stable and tissue-specific in specific diseases [92]. Currently, various miRNAs have been reported to play vital role in the pathogenesis of DKD (Table 2) [93]. These miRNAs can be divided into T1DN-related miRNAs, T2DN-related miRNAs, and not-mentioned types. Due to the lack of in-depth research, some miRNAs, such as miR-4490[94]and miR-423-5p[95], is only limited to in vitro level. In this section, we will mainly focus on the regulatory role of miRNAs on T1DN and T2DN.

**Table 2:** miRNAs involved in diabetes-related renal inflammation and fibrogenesis

Types	Functions involved	miRNAs	Expression levels	Target genes	Mechanisms	Ref
T1DN-related miRNAs	Renal Inflammation	miR-21	Upregulation	TIMP3	upregulation of miR-21 enhanced the excretion of pro-inflammatory factors by repressing the expression of TIMP3	[96]
		miR-146a	Upregulation	IRAK1/TRAF6	upregulation of miR-146a promoted NF-kB mediated upregulation of pro-inflammatory cytokines by negative feedback to Irak1 and Traf6	[97]
		miR-146a	Downregulation	Nox4	miR‑146a/Nox4 decreases ROS generation and inflammation and prevents DN	[98]
	Renal Fibrosis	miR-192	Upregulation	GLP1R	upregulation of miR-192 exerted its pro-fibrotic effects by directly targeting GLP1R	[99, 100]
		miR-214	Upregulation	PTEN	upregulation of miR-214 contributed to renal cell hypertrophy and matrix protein expression by directly acting on PTEN	[101]
		miR-22	Upregulation	PTEN	upregulation of miR-22 promoted renal tubulointerstitial fibrosis by suppressing autophagy partially via targeting PTEN	[102]
		miR-382	Upregulation	FOXO1	upregulation of miR-382-induced glomerular mesangial cell proliferation and ECM accumulation by targeting FOXO1	[103]
		miR-137	Downregulation	Notch1	miR-137 inhibition aggravated ECM protein accumulation via directly targeting Notch1	[104]
	Both inflammation and fibrosis	miR-455-3p	Downregulation	ROCK2	downregulated miR-455-3p aggravated the progression of renal inflammation and fibrosis through promoting ROCK2 expression	[105]
T2DN related miRNAs	Renal Inflammation	miR-146a	Upregulation	IRAK1/TRAF6	upregulation of miR-146a promoted NF-kB mediated upregulation of pro-inflammatory cytokines by negative feedback to Irak1 and Traf6	[97]
	Renal Fibrosis	miR-133b/199b	Upregulation	Sirt 1	upregulation of miR-133b and miR-199b enhanced TGF-β1-induced epithelial to mesenchymal transition and renal fibrosis by targeting SIRT1 in diabetic nephropathy	[107]
		miR-23a	Upregulation	SnoN	upregulation of miR-23a promoted high glucose-induced EMT and renal fibrogenesis by down-regulation of SnoN	[112]
		miR-30e	Downregulation	GLIPR-2	miR-30e inhibited GLIPR-2 and then promoted the proliferation of RTECs and inhibited EMT, ultimately leading to renal fibrosis in DN	[109]
		miR-93	Downregulation	Orai1	downregulation of microRNA-93-induced TGF-b1-induced EMT and renal fibrogenesis by down-regulation of Orai1	[110]
	Both inflammation and fibrosis	miR-29b	Downregulation	SP1/Smad-3/NF-κB	miR-29b played a protective role in diabetic kidney disease by the inhibition of Sp1 expression, TGF-β/Smad3-dependent renal fibrosis, and NF-κB-driven renal inflammation	[106]
	Renal Inflammation	miR-423-5p	Downregulation	Nox4	miR-423-5p suppressed high-glucose induced podocyte injury and inhibited ROS generation by targeting Nox4	[95]
Not mentioned	Renal Fibrosis	miR-4490	Upregulation	PSMA6	upregulation of miRNA-4490 regulated PSMA6 mRNA level post-transcriptionally	[94]
		miR-326-3p	Downregulation	FcγRIII	miR-326-3p ameliorates high glucose and ox-LDL-IC-induced fibrotic injury in renal mesangial cells by targeting FcγRIII	[113]
	Both inflammation and fibrosis	miR-199a-5p	Upregulation	Klotho	upregulated expression of miR-199a-5p decreased Klotho expression, resulting in activating the TLR4/NF-kB p65/NGAL signaling pathways and the downstream fibrosis and inflammation in HG-induced rat mesangial cells	[108]

#### T1DN-related miRNAs

In STZ-induced type 1 diabetic nephropathy mice models, multiple miRNAs have emerged as important regulators in the inflammatory responses and fibrogenesis of DKD. In in vitro and in vivo studies, the upregulated expression of miR-21 is able to enhance the excretion of pro-inflammatory factors and accelerate kidney injury by repressing the expression of tissue inhibitors of metalloproteinase 3, and thereby promote inflammation [96]. Conversely, miR-146a downregulates nicotinamide adenine dinucleotide phosphate oxidase 4 (Nox4) and its downstream activities of inflammatory cytokines, such as IL-1b and IL-18 [97, 98]. miR-192 was the first miRNA which was shown to have functional role in promoting the expression of ECM and collagens in STZ-induced mice [99, 100]. In the hyperglycemic environment, the elevated miR-214 and miR-22 were found to be involved in renal fibrogenesis both by inhibiting the expression of phosphatase and tensin homolog (PTEN) [101, 102]. Many other miRNAs, such as miR-382 and miR-137, were also reported to participate glomerular and tubular fibrogenesis of T1DN [103, 104]. In addition, the overexpression of miR-455-3p in STZ-induced DN rats cannot only improve renal fibrosis but reduce inflammatory cytokines by targeting rho-associated coiled coil-containing protein kinase 2 (ROCK2) [105]. Therefore, dysregulation of miRNAs plays catalytic roles in the progression of T1DN inflammation and fibrosis.

#### T2DN-related miRNAs

Numerous miRNAs are now also thought to be involved in the progression of type 2 diabetic nephropathy. Among these miRNAs, miR-146a is the only one which also exert anti-inflammatory role on the pathogenesis of diabetic db/db mice[97]. However, in the T2DM experimental models, studies on the regulatory role of miRNA mainly focus on the processes of fibrogenesis. Among the miR-29 family, decreased miR-29b, not miR-29a and c, is demonstrated to ameliorate both renal inflammation and fibrogenesis. Loss of intrarenal miR-29b is able to inhibiting the progression of DKD via TGF-β/Smad3-dependent renal fibrosis and NF-κB-driven renal inflammation [106]. Mechanistically, as downstream targets of TGF-β signaling, miR-133b and miR-199b dramatically facilitate TGF-β1-induced EMT & renal fibrosis by inhibiting the expression of sirt1 in diabetic OLETF rats [107]. In addition, there is evidence that high glucose activates TLR4/NF-κB and p65/NGAL signaling pathways by upregulating miR-199a, along with the reduction of klotho, resulting in both fibrosis and inflammatory reaction [108]. Apart from miR-29b and miR-199, miR-30e [109], miR-93 [110, 111], miR-23a [112], and miR-326-3p [113] can affect the synthesis of ECM protein in MCs and the EMT by regulating their target genes, resulting in the excessive accumulation of ECM.

### The role of LncRNAs in diabetes-related renal inflammation and fibrosis

**Fig. 2:**
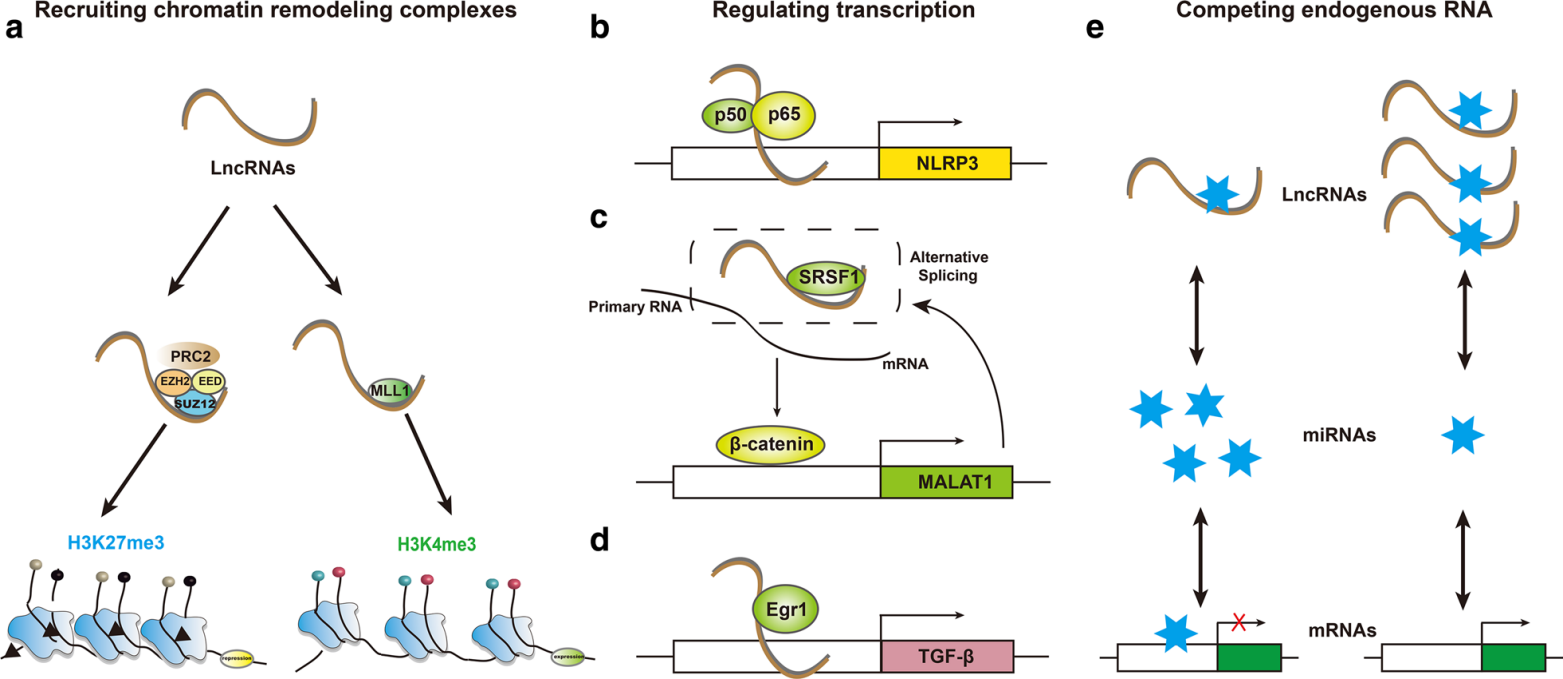
Function of LncRNAs in diabetes-related renal inflammation and fibrosis. LncRNAs can regulate the expression of adjacent and distal genes by various biological mechanisms in DKD. As shown above, **a** LncRNAs promote the binding of H3K4me3 or H3K27me3 to the gene promoter to affect its expression via recruiting chromatin remodeling complexes such as PRC2 and MLL1. LncRNAs bind transcription factors or cofactors to affect the transcription of target genes as scaffolds: **b** LncRNAs regulate NLRP3 inflammasome signaling pathway by interacting with p50, the subunit of NF-κB; **c** LncRNA MALAT1 accelerates β-catenin nuclear accumulation through physical binding to SRSF1, and thus feedback to promote the expression of LncRNA MALAT1 and contribute to renal fibrosis; **d** LncRNAs also act as regulators of inflammation via directly interaction with Egr-1. In addition, **e** LncRNAs located in the cell cytoplasm regulate gene expression by acting as molecular sponges and competitively binding to miRNA. EED, embryonic ectoderm development; PRC2, polycomb repressive complex 2; SUZ12, PRC2 subunit; MLL1, mixed-lineage leukemia 1; Egr‐1, early growth response protein 1

Unlike miRNA, the expression of LncRNAs has poor sequence conservation among different species and is highly specific in different tissues and cell types; thus, the in-depth study of different LncRNAs could improve our understanding of the metabolic memory mechanisms of DKD. As an integral part of the epigenome, LncRNAs regulate the expression of adjacent and distal genes expression by various biological mechanisms, which include recruiting chromatin remodeling complexes, regulating gene expression as competitive endogenous RNA, and binding transcription factors or cofactors to affect the transcription of target genes as a scaffold [114, 115]. Lately, accumulating novel data have been instrumental in identifying potential biological functions of LncRNAs that contribute to renal inflammation and fibrogenesis associated with both T1DN and T2DN (Fig. 2).

#### Recruitment of chromatin remodeling complex

Previous studies have demonstrated that LncRNAs can mediate epigenetic changes by recruiting chromatin remodeling complexes to specific gene promoters in various diseases, including DKD [116, 117]. A 2018 study indicates that LINC00968 promotes the binding of H3K27me3 to the p21 promoter to inhibit its expression via the recruitment of EZH2, a vital subunit of PRC2 complex. The silencing of LINC00968 significantly inhibited the proliferation and fibrosis of MCs, and decreased the expression of ECM proteins such as fibronectin and collagen IV in vitro. [118]. Moreover, directed by their related LncRNAs, this model is also applicable to other chromatin modifying complexes, such as methyltransferase myeloid and lymphoid or mixed-lineage leukemia (MLL), PcG and so on. LncRNA ZEB1-AS1, which is the antisense lncRNA located in the promoter region of zinc finger E-box-binding protein 1 (ZEB1) and positively regulates its expression, displayed an anti-fibrotic role in DKD by reducing the high glucose-induced ECM accumulation. Mechanistically, LncRNA ZEB1-AS1 directly binds H3K4 methyltransferase MLL1 and induces H3K4me3 histone modification in ZEB1 promoter, which further activates ZEB1 transcription. Furthermore, the expression of lncRNA ZEB1-AS1 can be suppressed by p53 via the physical interaction with its promoter region, highlighting intricate epigenetic regulatory mechanisms in both T1DN and T2DN murine models [119].

#### Binding transcription factors or cofactors

Emerging studies also hint to the central role of LncRNAs in regulating transcription and post-transcription of fibrosis-associated and inflammation-related genes in DKD. Among 12 abnormally expressed LncRNAs, LincRNA-Gm4419 is verified to be the only one related to NF-κB by bioinformatic methods. To further determine its role on the pathogenesis of DKD, the deletion of LincRNA-Gm4419 by small interfering RNAs (siRNA) could significantly decrease the inflammation, fibrosis, and cell proliferation. The pro-inflammatory effect of LincRNA-Gm4419 is mediated through direct interaction with p50, the subunit of NF-κB and NLRP3 inflammasome signaling pathway [120]. LncRNA MALAT1, as a broadly expressed nuclear lncRNA in mammalian tissues, is found highly expressed not only in STZ-induced diabetic C57BL/6 mice, but also in high glucose-induced podocytes. SRSF1, also known as SF2/ASF, is one of the major pre-mRNA splicing factors that contribute to partial RNA metabolism, such as nuclear export of the mature mRNA [121]. In Lv’s study [122], LncRNA MALAT1 has been shown to accelerate β-catenin nuclear accumulation through physical binding to SRSF1, and thus promote podocyte injury and contribute to renal fibrosis. Moreover, recent studies demonstrate that LncRNA Rpph1 and LncRNA NONHSAG053901 acts as regulators of inflammation via the directly interaction with the DKD-related factor galectin-3 (Gal-3), or early growth response protein 1 (Egr‐1), respectively [123, 124]. In addition, Egr‐1 is able to augment its function in DKD inflammation by interacting with TGF‐β, and the overexpression of Egr-1 can also upregulate the lncRNA Arid2-IR level which contributes to ECM accumulation in DKD [125], in support of a complicated potential mechanism of metabolic memory in DKD.

#### Crosstalk between LncRNA and miRNAs

As previously mentioned, LncRNAs located in the cell cytoplasm, which act as molecular sponges, also involved in the development of renal inflammation and fibrosis through competitively binding to miRNA. Erbb4-IR, a smad3-associated profibrotic lncRNA, enhances the progression of renal fibrosis and increases albuminuria in DKD by inhibiting miR-29b in the transcriptional level. An in vitro study also revealed that the elevated expression of LncRNA Erbb4-IR was induced by AGEs rather than by high glucose [126]. A novel LncRNA, MEG3, is upregulated and promotes cell fibrosis and inflammatory in response to DKD by suppressing miR-181a and targeting the aforementioned genes, *Egr-1* and *TLR4*. The interaction of LncRNA MEG3 with miR-181 is not only through sponging by competing with endogenous RNA mechanisms, but direct targeting by and in an Argonaute (Ago)2-dependent manner [127]. Ago2 is a major member of the RNA-induced silencing complexes (RISCs), which play a central role in post-transcriptional gene regulation. miRNAs direct complementary target mRNAs to repress and/or degrade RNA through RISCs that contains Ago family proteins [128]. The down-regulation of another lncRNA, LincRNA1700020I14Rik, promotes MCs proliferation and fibrosis in DKD through miR-34a-5p via directly targeting way and Ago2-dependent manner. Additionally, LincRNA 1700020I14Rik/miR-34a-5p/ sirt1 axis and LncRNA-NR_033515/ miR-743b-5p axis were also found to be involved in fibrogenesis of DKD [129, 130]. In any case, these findings provide a better understanding of the role of LncRNAs in inflammatory and fibrogenic progression and may lead to novel therapies for DKD.

### The role of circRNAs in diabetes-related renal inflammation and fibrosis

CircRNAs are a novel class of non-coding RNAs, which are mainly generated from the back-splicing of exons, introns or a combination of both, forming a covalently closed loop [131]. In the inflammatory and fibrogenic processes of both T1DN and T2DN, circRNAs canonically function as a competing endogenous RNA to protect the target genes from miRNA‐mediated mRNA degradation, thereby achieving their biological role (Table 3).

**Table 3:** circRNAs involved in diabetes-related renal inflammation and fibrogenesis

CircRNAs	Experimental models	Functions involved	Axis	Ref
circRNA_15698	T2DN, Diabetic db/db mice	ECM accumulation	circRNA_15698/miR-185/TGF‐β1 axis	[132]
circRNA-0080425	T1DN, STZ-induced mice	renal fibrosis	circRNA-0080425/miR‐24‐3p/FGF11 axis	[133]
circ-LRP6	Not mentioned, mesangial cells	renal inflammation and fibrosis	circLRP6/miR‐205/HMGB1 axis	[134]
circ-WBSCR17	T1DN, STZ-induced mice	inflammatory responses and fibrosis	circ-WBSCR17/miR-185-5p/SOX6 axis	[135]
circ-RNF169	Not mentioned, glomerular endothelial cells	cell proliferation and EMT	Unknown	[136]
circSTRN3	Not mentioned, glomerular endothelial cells	cell proliferation and EMT	Unknown	[136]

With the help of circRNA microarray analysis, circRNA_15698 was found to be upregulated in both diabetic db/db murine models and in the HG-induced MCs. The overexpression of circRNA_15698 play pivotal role in ECM accumulation via miR‐185/TGF‐β1 axis [132]. Another novel circRNA, 0080425, significantly accelerates cell proliferation and fibrosis by suppressing the expression of miR‐24‐3p. The inhibition of this miRNA releases its suppression on fibroblast growth factor 11 (FGF11), which stimulate endothelial proliferation and migration in STZ-induced T1DN [133]. Finally, the circLRP6 upregulates the expression of high mobility group box 1 (HMGB1) via the circLRP6-miR‐205-HMGB1 regulatory network, thus ameliorates HG‐induced MCs proliferation, ECM accumulation, and renal inflammation [134]. In high glucose-induced human kidney tubular cells, Circ-WBSCR17 was also reported to aggravate inflammatory responses and fibrosis by targeting miR-185-5p/SOX6 regulatory axis[135]. CircRNF169 and circSTRN3 are another two differentially expressed circRNAs that were found to be involved in HG-induced cell proliferation and EMT [136]. However, investigating their undefined mechanisms will provide novel insights for the biological role of circRNAs associated with renal inflammation and fibrosis.

## Conclusion

The present review focused on the biologic role of metabolic memory, mainly in pathological processes, such as inflammation and fibrosis and potential treatment strategies, especially epigenetic changes-oriented therapies, of DKD. In short, epigenetic mechanisms that include DNA methylation, histone PTMs, and ncRNAs play critical roles in the pathogenesis of DKD, especially in the processes of inflammation and fibrogenesis. Actually, these mechanisms are not completely independent. DNA methylation can interact with histone modification, which in turn can be affected by miRNAs and LncRNAs. Once triggered by high glucose stimulation or other diabetic pathogenic factors, these intricate interactions of epigenetic changes can persistently activate pro-inflammatory and fibrotic genes in both T1DN and T2DN. Despite subsequent glycemic control, the existence of metabolic memory mediated by epigenetic mechanisms leads to the development of renal inflammation and fibrosis, and then progress to ESRD. These evidences hereinbefore demonstrate persistent epigenetic changes (DNA methylation and histone modifications) and signaling pathway mediated by non-coding RNAs may be involved in metabolic memory. In addition, the constant epigenetic changes can be transferred to offsprings, and affect the population phenotype within a very short period of time.

Therefore, in addition to intensifying blood glucose control in the early stages of diabetes, and reducing the long-term effects of hyperglycemic "metabolic memory," reasonable and effective treatment measures should be taken according to the above epigenetic mechanisms. Various epigenetic drugs, such as DNMT and HDAC inhibitors, are currently reported to play beneficial effects in preclinical studies. For ncRNAs, locked nucleic acid (LNA)-modified oligonucleotides based on antisense technology present superior sensitive and specific detecting abilities because of its high thermal stability when hybridized with their complementary mRNA. Antisense technology has been administrated successfully to targets LncRNAs in many other diseases other than DKD. In the context of diabetes, however, the administration of LNA-modified miRNA inhibitors was demonstrated to ameliorate the renal damage of DKD, especially renal fibrosis. Identifying the precise therapeutic target for antisense therapy in DKD is urgently needed, which is also the aim of this review. Unlike genetics, epigenetics is mostly reversible, which provides a new therapeutic frontier for DKD prevention and management. Thus, more researches on epigenetics and the development of epigenetic-modifying drugs are required in the future, so as to provide reliable biomarkers and novel therapeutic targets for the progression of DM to DKD by altering epigenetics.

## Data Availability

No additional supporting data in this article.

## Abbreviations

AGEs: Advanced glycation end products
ADP: Adenosine diphosphate
ANGPTL2: Angiogenin-like protein 2
Ago: Argonaute
CCL2: Chemokine (C–C motif) ligand 2
CTGF: Connective tissue growth factor
CircRNAs: Circular RNAs
DKD: Diabetic kidney disease
DM: Diabetes Mellitus
DCCT: Diabetes Control and Complications Trial
DMR: Differentially methylated region
DNMTs: DNA methyltransferases
EDIC: Epidemiology of diabetes interventions and complications
ECM: Extracellular matrix
eGFR: Estimated glomerular filtration rate
ESRD: End-stage renal disease
Egr‐1: Early growth response protein 1
ER: Endoplasmic reticulum
FGF11: Fibroblast growth factor 11
Gal-3: Galectin-3
HATs: Histone acetyltransferase
HDACs: Histone deacetylase
HMTs: Histone lysine methyltransferase
HDMs: Histone lysine demethylase
HMGB1: High mobility group box 1
HbA1c: Average glycated hemoglobin
HG: High glucose
ICAM1: Intercellular adhesion molecule 1
IL: Interleukin
JAK-STAT: Janus kinase/signal transducers and activators of transcription
Kme1: Monomethylation
Kme2: Demethylation
Kme3: Trimethylation
LNA: Locked nucleic acid
LncRNAs: Long non-coding RNAs
miRNAs: MicroRNAs
MLL: Mixed-lineage leukemia
MCs: Mesangial cells
MMP: Matrix metalloproteinases
5-mC: 5-Methylcytosine
ncRNAs: Non-coding RNA
Nox4: Nicotinamide adenine dinucleotide phosphate oxidase 4
Nrf2-keap1: NF-E2–related factor 2/ Kelch-like ECH associated protein 1
NAD +: Nicotinamide adenine dinucleotide
NGS: Next-Generation Sequencing
PTEN: Phosphatase and tensin homolog
PAI-1: Plasminogen activator inhibitor-1
PXR: Pregnane X receptor
PTMs: Post-translational modifications
ROS: Reactive oxygen species
Rgc32: Response gene to complement 32
RISCs: RNA-induced silencing complexes
ROCK2: Rho-associated coiled coil-containing protein kinase 2
Sirt: Sirtuins
siRNA: Small interfering RNAs
STZ: Streptozocin
SAM: S-adenosylmethionine
SAH: S-adenosyl homocysteine
TSA: Trichostatin A
TXNIP: Thioredoxin interacting protein
TET: Ten-eleven translocation
TGF-β: Transforming growth factor-β
TNF-α: Tumor necrosis factor-α
UTR: Untranslated regions
VCAM1: Vascular cell adhesion molecule 1
UKPDS: UK Prospective Diabetes Study
ZEB1: Zinc finger E-box-binding protein 1
